# Community engagement in WHO guideline development

**DOI:** 10.2471/BLT.24.291579

**Published:** 2024-09-10

**Authors:** Manjulaa Narasimhan, Patricia Mahecha Gutiérrez, Zoë Osborne, Muluba Habanyama, Karrie Worster, Carrie Martin, Angela Kaida

**Affiliations:** a Department of Sexual and Reproductive Health and Research, World Health Organization, Avenue Appia 20, 1211 Geneva 27, Switzerland.; b Research Institute of the McGill University Health Centre, Quebec, Canada.; c Faculty of Health Sciences, Simon Fraser University, Burnaby, Canada.; d Ontario HIV Treatment Network, Toronto, Canada.; e Indigenous Health Centre of Tiohtià:ke, Montreal, Canada.

The development of World Health Organization (WHO) guidelines is multidisciplinary and includes input from a range of experts such as policy-makers, programme managers, civil society and health workers in the elaboration of evidence-based recommendations. WHO also urges guideline developers to include input of individuals and communities who are affected by the recommendations. Community engagement, which is different from civil society engagement, often means engaging meaningfully with marginalized and underserved individuals most affected by but not often involved in developing normative recommendations. Community engagement in the development of normative guidelines necessitates time, commitment and funding to develop relationships characterized by respect, trust and purpose, enabling stakeholders to work together to achieve more equitable health and social outcomes.^1^

WHO recommendations that consider the voices of affected individuals and communities can better reflect the realities of the intended beneficiaries of normative guidelines and thus provide them with the best chance of improved health and well-being. Furthermore, community engagement can facilitate in-country implementation of global guidelines as well as support translating community research into global policy reform for national action.^2^ Here we describe community engagement in the development and adoption at national level of the WHO *Consolidated guideline on sexual and reproductive health and rights of women living with HIV*.^3^

Development of this WHO guideline was grounded in community engagement at every step in the process, thereby comprehensively integrating the input of women living with human immunodeficiency virus (HIV) in all their diversity. This process started with a global values and preferences survey in 2015, developed by and for women living with HIV, that collated the needs, perspectives and priorities of women living with HIV from 94 countries.^4^^,^^5^ As a result of survey findings, the guideline gives prominence to safety, respect and support for women’s physical and mental well-being, ensuring a woman-centred and human rights-based approach across the life-course for all women living with HIV.^6^

The subsequent work of implementing partners suggests that successful adoption and contextualization of the guideline is tied directly to the depth and quality of community involvement in implementation efforts.^7^ The success of engaging with underserved communities from the start of guideline development led to the WHO Director-General in April 2019 establishing WHO’s first and so far only community advisory group made up entirely of underserved communities – the WHO Advisory Group of Women Living with HIV. In addition, including the values and preferences of affected end-users, individuals and communities became core to all WHO guideline development.

A direct example of the inclusive, collaborative approach in the development of the guideline is a five-year implementation study supporting the strengthening of research capacity of Indigenous women, girls and gender-diverse individuals living with HIV in Canada, Guatemala, India, Nepal, New Zealand, Nigeria and Peru that is being conducted in partnership with local Indigenous organizations. Engaging these organizations and Indigenous communities from the start of guideline implementation research acknowledged the value of their knowledge, perspectives and experiences in advancing sexual and reproductive health rights outcomes. This engagement, in turn, fostered a sense of ownership and empowerment for ensuring that health-care interventions related to sexual and reproductive health rights are culturally sensitive, contextually appropriate and socially inclusive.

Early anecdotal evidence from these seven countries suggests high adherence to the guideline by local health workers, improved sexual and reproductive health and rights outcomes among Indigenous women and girls, and overall acceptance of the guideline’s recommendations by the communities. Furthermore, the engagement of Indigenous community leaders and youth underscored the intergenerational nature of community empowerment and knowledge transmission, ensuring that efforts to improve health-care outcomes are sustainable and rooted in local wisdom and traditions.^8^ National consultations and webinars reveal that community engagement fostered a sense of solidarity, resilience and self-determination among Indigenous populations, paving the way for more sustainable and equitable health systems that prioritize the needs and rights of Indigenous women living with HIV and their communities. The ongoing engagement in these seven countries has also allowed for a continuous feedback loop, ensuring that the guideline remains relevant and the global recommendations are effectively implemented.

**Fig. 1:**
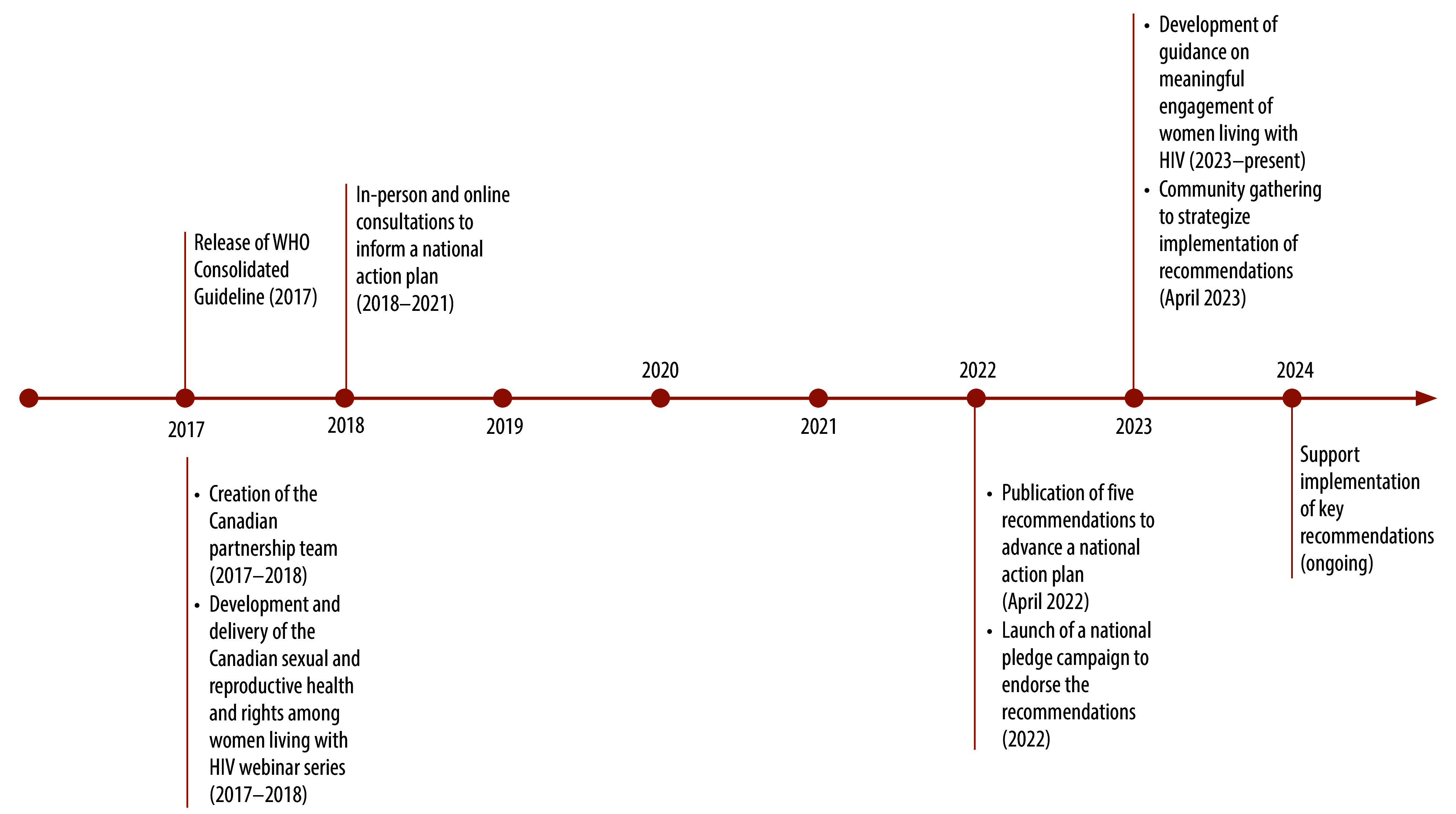
Phases for developing and implementing recommendations to inform a national action plan to advance sexual and reproductive health and rights of Women Living with HIV, Canada (2017–present)

HIV: human immunodeficiency virus; WHO: World Health Organization.

The inclusive approach of community engagement before, during and after the development of the guideline has also facilitated partnerships to support national implementation of the guideline. Beginning in 2017, leading HIV community, research and clinical organizations across Canada partnered with the WHO Department of Sexual and Reproductive Health to implement the guideline and develop recommendations for a national action plan on sexual and reproductive health rights, focused on creating enabling environments for women living with HIV.^9^ This partnership included a global webinar series to define priorities in sexual and reproductive health rights among women living with HIV in Canada, disseminate research conducted in Canada and best practices to demonstrate the central importance of meaningful engagement of women living with HIV in all efforts to advance health research, care and policy. The webinar series was structured to align with recommendations and good practice statements for creating and maintaining enabling environments and supporting health interventions outlined in the global guideline, while prioritizing topics relevant to the Canadian context. Driven by priorities of women living with HIV in Canada and informed by a national research evidence base, the webinar series highlighted four topics: (i) trauma and violence-aware care and/or practice; (ii) supporting safer HIV disclosure; (iii) reproductive health, rights and justice; and (iv) resilience, self-efficacy and peer support. Building on learnings from the webinar series alongside scientific evidence from a national women and HIV research programme, a multidisciplinary team of women living with HIV, researchers, community advocates, global and national policy-makers, and clinical and social service providers conducted national consultations to establish five key recommendations for a national action plan to advance the sexual and reproductive health and rights of women living with HIV in Canada (Fig. 1).^10^

Table 1 shows specific recommendations for one of the four prioritized webinar topics on supporting safer HIV disclosure that emerged from the community-engaged processes and discussions in Canada, and highlights how the national recommendations align with WHO global recommendations and good practice statements outlined in the guideline.

**Table 1:** Alignment of the five key recommendations for a national action plan to advance the sexual and reproductive health and rights of women living with HIV in Canada with WHO good practice statements and recommendations^a^ regarding supporting safer HIV disclosure

Key recommendations	Topic-specific recommendations in the Canadian National Action Plan for the prioritized topic of supporting safer HIV disclosure^b^	WHO good practice statements^a^ aligned with the topic-specific recommendations	Aligned WHO recommendations^a^
1. Meaningfully engage women living with HIV across research, policy and practice aimed at advancing the sexual and reproductive health and rights by, with and for all women living with HIV	1.1 HIV disclosure is a personal and individual choice; safety looks different for each person, and therefore the individual decision of how, when and to whom to disclose should be supported. 1.2 Intersections of race, culture, class, gender identity and sexual orientation shape disclosure and safety; acknowledgement of such structural and systemic inequities can inform effective policy and programming. 1.3 Integrate social networks and peer systems into existing supports for women living with HIV who are navigating disclosure.	**Psychosocial support** Statement 1: Psychosocial support interventions, such as support groups and peer support, provided by, with, and for women living with HIV, should be included in HIV care. **Healthy sexuality across the life course** Statement 2: Women living with HIV in all their diversity should be supported in their choice to have safe and fulfilling sexual relationships and sexual pleasure as they age. Women living with HIV who choose not to be sexually active should also be supported in their choice. **Integration of SRHR and HIV services** Statement 5: Women living with HIV should be included in the design and delivery of integrated and tailored comprehensive sexual and reproductive health and HIV services. **Social inclusion and acceptance** Statement 9: Policy-makers, parliamentarians and other public health leaders should work together with civil society organizations in their efforts to monitor stigma, confront discrimination against key populations and change punitive legal and social norms.	**Sexual health counselling and support** Recommendation 1: WHO recommends that for women living with HIV, interventions on self-efficacy and empowerment around sexual and reproductive health and rights should be provided to maximize their health and fulfil their rights. **Violence against women services** Recommendation 4: WHO recommends that policy-makers and service providers who support women living with HIV who are considering voluntary HIV disclosure should recognize that many fear, are experiencing or are at risk of intimate partner violence. Recommendation 5: WHO recommends that interventions and services supporting women living with HIV who are considering voluntary HIV disclosure should include discussions about the challenges of their current situation, the potential associated risk of violence and actions to disclose more safely, and facilitate links to available violence prevention and care services. Recommendation 6: Adolescents should be counselled about the potential benefits and risks of disclosure of their HIV status to others and empowered and supported to determine if, when, how and to whom to disclose. Recommendation 7: HIV testing services for couples and partners, with support for mutual disclosure, should be offered to individuals with known HIV status and their partners.
2. Centre Indigenous women’s priorities, voices and perspectives in all efforts to advance sexual and reproductive health and rights of women living with HIV	2.1 Indigenous-led and culturally safe supports must be available to Indigenous women living with HIV as they navigate safer HIV disclosure. Such supports should respond to the unique needs of Indigenous women living with HIV and should be grounded in culturally relevant approaches (such as through incorporation of cultural ceremony or support from Elders, as appropriate).	**Integration of SRHR and HIV services** Statement 4: Women living with HIV should have access to integrated and tailored comprehensive sexual and reproductive health (SRH) and HIV services. **Social inclusion and acceptance** Statement 10: Health-care workers should receive appropriate recurrent training and sensitization to ensure that they have the skills, knowledge and understanding to provide services for adults and adolescents from key populations based on all persons’ right to health, confidentiality and non-discrimination. **Community empowerment** Statement 12: Programmes should be put in place to provide legal literacy and legal services to key populations so that they know their rights and applicable laws and can receive support from the justice system when aggrieved.	
3. Use language and terminologies that are actively destigmatizing, inclusive and reflective of women living with HIV’s strengths and experience when discussing sexual and reproductive health and the rights of women living with HIV	3.1 Intentional shifts in language used to discuss HIV disclosure are essential; for example, the term disclosure is closely tied to the legal system and its continued use may emphasize the legal aspects of HIV disclosure over individual, spiritual, cultural and relational aspects.	**Social inclusion and acceptance** Statement 9: Policy-makers, parliamentarians and other public health leaders should work together with civil society organizations in their efforts to monitor stigma, confront discrimination against key populations and change punitive legal and social norms. Statement 10: Health-care workers should receive appropriate recurrent training and sensitization to ensure that they have the skills, knowledge and understanding to provide services for adults and adolescents from key populations based on all persons’ right to health, confidentiality and non-discrimination.	
4. Strengthen and expand knowledge translation initiatives to support access to and uptake of relevant and contemporary sexual and reproductive health and rights information for all stakeholders	4.1 Ensure that women living with HIV have the tools and resources needed to support safer HIV disclosure, including accurate, up-to-date, evidence-informed information about their health and rights. 4.2 HIV-related stigma threatens the safety of women living with HIV, particularly regarding HIV disclosure. This risk is actively challenged by developing and implementing robust knowledge translation efforts that target diverse audiences. Such efforts should be appropriate, applicable and adaptable to various settings and sectors; for example, knowledge translation efforts should be used to train and educate service providers to maintain confidentiality and promote safer service environments for women living with HIV.	**Protection from violence and creating safety** Statement 6: Violence against people from key populations should be prevented and addressed in partnership with key population-led organizations. All violence against people from key populations should be monitored and reported, and redress mechanisms should be established to provide justice. Statement 8: Law enforcement officials and health- and social-care providers need to be trained to recognize and uphold the human rights of key populations and to be held accountable if they violate these rights, including perpetration of violence. **Social inclusion and acceptance** Statement 9: Policy-makers, parliamentarians and other public health leaders should work together with civil society organizations in their efforts to monitor stigma, confront discrimination against key populations and change punitive legal and social norms Statement 10: Health-care workers should receive appropriate recurrent training and sensitization to ensure that they have the skills, knowledge and understanding to provide services for adults and adolescents from key populations based on all persons’ right to health, confidentiality and non-discrimination. **Community empowerment** Statement 12: Programmes should be put in place to provide legal literacy and legal services to key populations so that they know their rights and applicable laws and can receive support from the justice system when aggrieved.	Recommendation 8: Initiatives should be put in place to enforce privacy protection and institute policy, laws and norms that prevent discrimination and promote tolerance and acceptance of people living with HIV. This can help create environments where disclosure of HIV status is easier.
5. Catalyse the reciprocal relationship between evidence and action such that action on sexual and reproductive health and rights is guided by research evidence, and research is guided by what is needed for effective action	5.1 Current laws in Canada that criminalize the non-disclosure of HIV to sexual partners threaten the health and safety of women living with HIV; such laws must be updated to reflect the current scientific understanding of HIV transmission, which indicates that either viral suppression or condom use *alone* are sufficient to prevent the sexual transmission of HIV.	**Integration of sexual and reproductive health and rights and HIV services** Statement 4: Women living with HIV should have access to integrated and tailored comprehensive sexual and reproductive health (SRH) and HIV services. **Protection from violence and creating safety** Statement 6: Violence against people from key populations should be prevented and addressed in partnership with key population-led organizations. All violence against people from key populations should be monitored and reported, and redress mechanisms should be established to provide justice. Statement 7: Health and other support services should be provided to all persons from key populations who experience violence. Persons experiencing sexual violence should have timely access to comprehensive post-rape care in accordance with WHO guidelines. **Supportive laws and policies and access to justice** Statement 13: Countries should work towards decriminalization of behaviours such as drug use/injecting, sex work, same-sex activity and nonconforming gender identities, and towards elimination of the unjust application of civil law and regulations against people who use/inject drugs, sex workers, men who have sex with men and transgender people.	

HIV: human immunodeficiency virus; SRH: sexual and reproductive health; SRHR: sexual and reproductive health and rights; WHO: World Health Organization; .

^a^ From WHO, 2017.^3^

^b^ Additional details about the topic-specific recommendations are available as a supplementary material file in Kaida et al., 2022.^9^

Note: The good practice statements and recommendations are verbatim from the WHO *Consolidated guideline on sexual and reproductive health and rights of women living with HIV*.^3^

Although initially delayed by the coronavirus disease 2019 (COVID-19) pandemic, implementation activities restarted in 2022. To promote uptake of the recommendations, the Canada leadership team developed an online endorsement pledge and in 2023, convened a national consultation to discuss strategies for implementation.^11^ This consultation reinforced the notion that meaningful engagement of women living with HIV (recommendation 1) is foundational for the implementation of all other recommendations in the Canadian national action plan. Partners identified principles for how to create the types of enabling environments that promote meaningful engagement of women living with HIV in all their diversity.^12^ This team continues to advance meaningful engagement and implementation of all five key recommendations within an accountability framework to promote the sexual and reproductive health and rights of women living with HIV in Canada. A forthcoming workshop in April 2025, inclusive of women living with HIV, will provide an opportunity for reaching consensus on how to monitor and evaluate uptake of the recommendations and determine community-informed indicators of implementation success. In its determination of success, the monitoring and evaluation plan will consider, for the global and national recommendations, accountability (did we implement the recommendations as planned?); impact (what are the impacts of implementing the recommendations?); and learning, development and adaptation (what is changing and how do we understand those changes to learn, adapt and develop strategies to improve the implementation recommendations?). This plan will broaden understanding on whether and how meaningful engagement of women living with HIV makes a difference to sexual and reproductive health rights programming, policy and research in Canada.

This work demonstrates some of the ways in which meaningful community engagement at every stage of guideline development can facilitate successful translation of global guidelines to national implementation. By predicating guidelines on the needs, perspectives and values of the people for whom the recommendations are intended, community engagement in guideline development promotes inclusivity and human rights approaches to health-care delivery, policies and programmes. 

For the continued success of initiatives that build partnerships and collaboration between global health institutions and grassroots organizations, funding community- and women-led initiatives is essential. In addition, guideline developers must take steps at the start of WHO guideline development to better understand how underserved individuals and communities navigate their rights within often complex health systems where power hierarchies undermine their rights. Community engagement fosters a sense of solidarity, resilience and self-determination, and is essential for paving the way for more sustainable and equitable health systems that prioritize the diverse and often invisible needs and rights of marginalized and underserved communities.
